# Development of a native-locus dual reporter system for the efficient screening of the hyper-production of natural products in *Streptomyces*

**DOI:** 10.3389/fbioe.2023.1225849

**Published:** 2023-06-29

**Authors:** Jing-Yi Zhou, Bin-Bin Ma, Qing-Wei Zhao, Xu-Ming Mao

**Affiliations:** ^1^ Department of Clinical Pharmacy, The First Affiliated Hospital and Institute of Pharmaceutical Biotechnology, School of Medicine, Zhejiang University, Hangzhou, China; ^2^ Zhejiang Provincial Key Laboratory for Microbial Biochemistry and Metabolic Engineering, Hangzhou, China; ^3^ Zhejiang Provincial Key Laboratory for Drug Evaluation and Clinical Research, Hangzhou, China

**Keywords:** *Streptomyces*, daptomycin, hyper-production, dual reporter, native locus, random mutagenesis, target screening

## Abstract

*Streptomyces* is renowned for its abundant production of bioactive secondary metabolites, but most of these natural products are produced in low yields. Traditional rational network refactoring is highly dependent on the comprehensive understanding of regulatory mechanisms and multiple manipulations of genome editing. Though random mutagenesis is fairly straightforward, it lacks a general and effective strategy for high throughput screening of the desired strains. Here in an antibiotic daptomycin producer *S. roseosporus*, we developed a dual-reporter system at the native locus of the daptomycin gene cluster. After elimination of three enzymes that potentially produce pigments by genome editing, a gene *idgS* encoding the indigoidine synthetase and a kanamycin resistant gene *neo* were integrated before and after the non-ribosomal peptidyl synthetase genes for daptomycin biosynthesis, respectively. After condition optimization of UV-induced mutagenesis, strains with hyper-resistance to kanamycin along with over-production of indigoidine were efficiently obtained after one round of mutagenesis and target screening based on the dual selection of the reporter system. Four mutant strains showed increased production of daptomycin from 1.4 to 6.4 folds, and significantly improved expression of the gene cluster. Our native-locus dual reporter system is efficient for targeting screening after random mutagenesis and would be widely applicable for the effective engineering of *Streptomyces* species and hyper-production of these invaluable natural products for pharmaceutical development.

## Introduction

*Streptomyces* belongs to the soil-dwelling actinomycetes and produces the most plentiful bioactive natural products (Quinn et al., 2020). It accounts for about two-thirds of the clinically used antibiotics and also is the main producer of many pharmaceutical agents, including immuno-suppressors, anti-cancer drugs, and enzyme inhibitors, etc. Moreover, the genome sequencing project reveals that *Streptomyces* usually contain 30–50 biosynthetic gene clusters (BGCs) to produce natural products (Nicault et al., 2020). However, only about 5%–10% of BGCs have been scientifically studied, and most of them are transcriptionally silenced in laboratory, which has prompted scientists to re-investigate *Streptomyces* for this rich repertoire of natural products by awakening these cryptic BGCs (Liu et al., 2021). There has always been an urgent demand to develop effective and efficient strategies to improve productivity, which is particularly important for the full understanding of biosynthetic mechanisms and the pharmaceutical development of these bioactive small molecules (Onaka, 2017).

The biosynthesis of these complex natural products are sequentially catalyzed by a cascade of enzymes, which are encoded by the genes within the corresponding BGCs. Thus, the efficient production of natural products in *Streptomyces* is extensively dependent on the catalysis of biosynthetic enzymes, expression of gene clusters, supply of building blocks, coenzymes, and energy (such as ATP, NAD(P)^+^/NAD(P)H) (Hertweck, 2009; Walsh, 2016), etc. A full understanding and transcriptional engineering of the regulatory networks are promising ways of improving the expression of biosynthetic enzymes, potentially enhancing the production of natural products in *Streptomyces* (Rigali et al., 2018). Nevertheless, the total regulatory networks might be more complex than we expect. For example, it has been found that cell morphology significantly affects the production of natural products (Khushboo et al., 2023). Moreover, target deciphering of the regulatory mechanisms for a certain BGC is highly technology-dependent and time-consuming. Heterologous expression of the whole BGCs in a universal cell chassis, along with promoter reconfiguration is emerging as an alternative promising way to visualize the cryptic natural products from *Streptomyces*. However, the efficient cloning and expression of giant BGCs from *Streptomyces* are challenges currently faced by the scientific community (Liu et al., 2018; Nepal and Wang, 2019; Xu and Wright, 2019). Alternatively, a cell-free system of *Streptomyces* is emerging as a new opportunity for natural product discovery and derivation, while some bottlenecks still need to be optimized (Moore et al., 2023). Moreover, the productivity of natural products from *Streptomyces* is not always satisfactory for downstream research and development (Kang and Kim, 2021).

Due to the regulatory complexity and ingenious balance of the whole cell metabolism, a small disturbance of the network will not always lead to ideal productivity. In contrast to the rational but partial reconfiguration of these regulatory networks, the random mutagenesis occurs through more overall alterations in the genome and is more straightforward as it is only based on the ultimate phenotypic outputs, for example, the productivity of natural products. It can be carried out with biological methods, such as insertion of mobile genetic elements transposons and random evolution by high resistance to antibiotics (Xu et al., 2017; Luo et al., 2018; Yu et al., 2019a), chemical agents, such as *N*-methyl-*N′*-nitro-*N*-nitrosoguanidine (MNNG) (Mo et al., 2013; Rubio et al., 2021) and more convenient physical measures mainly based on high-energy radiation from ultraviolet (UV) and the recently emerging atmospheric and room temperature plasma (ARTP) (Mo et al., 2013; Yu et al., 2019b; Ye et al., 2021), as well as the combinatory applications of above strategies (Mo et al., 2013; Yan et al., 2021). However, the biggest challenge of the mutagenesis strategy is the high throughput but efficient screening of strains for hyper-production of the desired natural products. To overcome this obstacle, many reporter systems have been developed in *Streptomyces*, such as chromogenic strategies with enzymes IdgS and XylE to produce the blue and yellow pigments (Li et al., 2015; Li et al., 2018), respectively, and antibiotic resistance to kanamycin (Li et al., 2018; Gongerowska-Jac et al., 2021). However, false positives frequently arise in single-reporter systems, because different disturbances might result in the same reporter phenotypes. For example, ribosome mutations might also lead to high resistance to kanamycin (Luo et al., 2018). Thus, dual reporter systems have been preferentially used to more efficiently obtain the desired mutants (Xiang et al., 2009). The reporter systems are usually designed linked to the target promoters for screening of improved transcriptional strength, but mostly on the replicative plasmids or integrated in the genome distant from the native promoters of the biosynthetic gene cluster (Gongerowska-Jac et al., 2021). Probably due to the influence of genomic locations or the incompleteness of transcriptional elements on the cloned promoter (Bilyk et al., 2017), the strength of the reporter system does not always correlate well with the productivity of natural products.

Daptomycin is a lipopeptide antibiotic produced by *Streptomyces roseosporus*. It is biosynthesized through the activation of decanoic acid and attachment to a cyclic peptide, which is catalyzed by DptEF and the non-ribosomal peptidyl synthetases (NRPSs) DptABCD. Genes *dptEFABCD* comprise a large operon to produce a giant polycistronic transcript for daptomycin biosynthesis (Miao et al., 2005; Baltz, 2009). Based on the genome mining of transcription factors and deciphering of regulatory mechanisms, the removal of the negative regulators (Mao et al., 2015; Mao et al., 2017; Luo et al., 2018) and stabilization of the positive pleiotropic regulator AtrA (Xu et al., 2023) have significantly increased daptomycin production. Meanwhile, targeting multi-level rational metabolic engineering by precursor supply, regulatory pathway reconstruction, byproduct removal, and gene cluster multiplicity have improved the yield by about 6.6 folds (Lyu et al., 2022). To more comprehensively disturb the regulatory networks for the efficient increase of daptomycin production, the present study developed a dual-reporter system at the native daptomycin gene cluster and efficiently obtained a strain with improved productivity of 6.4 folds with simple UV-induced mutagenesis. This strategy is highly effective for efficient screening and would be widely applicable in other *Streptomyces* species for the hyper-production of natural products.

## Methods

**Bacterial strains and growth conditions.** The bacterial strains used in this study are listed in Table 1. *Streptomyces roseosporus* L30 (wild type) (Sun et al., 2020) was used as the parental strain in this study. *Escherichia coli* DH5α was the host for plasmid construction, and ET12567/pUZ8002 was used for the conjugation of plasmids from *E. coli* into *S. roseosporus*. The wild type L30 and its derivative strains were cultured at 30°C with shaking at 250 rpm in 2% tryptic soy broth (TSB) liquid medium plus 5% PEG6000. *Streptomyces* strains were cultured at 30°C on the R5 solid medium for sporulation and the MS medium (2% mannitol, 2% soybean flour, 2% agar) for conjugation. Daptomycin production was carried out in the YEME medium containing 4% glucose, 0.5% peptone, 0.3% yeast extract and 0.3% malt extract for fermentation, and 0.1% decanoic acid (V: V = 1: 1 methyl oleate) was added every 12 h from 72 h to promote the biosynthesis of daptomycin (Sun et al., 2020). Kanamycin (Km) resistance screening of the dual reporter strains was performed on the yeast malt glucose (YMG) medium with 0.4% yeast extract, 1% malt extract, 0.4% glucose, 0.2% CaCO_3,_ and 2% agar. *E. coli* DH5α and *E. coli* ET12567/pUZ8002 were cultured in Luria-Bertani (LB) medium (1% tryptone, 0.5% yeast extract, 0.5% NaCl) at 37 °C.

**TABLE 1:** Strains used in this study.

Strains	Genotype or description	Reference
*S. roseosporus* L30	Wild type	Mao et al. (2015)
L31	*PKSII*, *melC*, *rppA* triple knock-out strain	This study
L32	*neo* and *idgS-sfp* integrated after *dptD* and *dptF*, respectively, in L31	This study
XM296	high-yield of daptomycin productivity after UV mutagenesis	This study
XM297	high-yield of daptomycin productivity after UV mutagenesis	This study
XM298	high-yield of daptomycin productivity after UV mutagenesis	This study
XM299	high-yield of daptomycin productivity after UV mutagenesis	This study
*E. coli* DH5α	Host for plasmid construction	Novagen
ET12567/pUZ8002	Conjugation host	Mao et al. (2015)

**Plasmid construction.** All plasmids and primers were provided in Table 2 and Supplementary Table S1, respectively. Plasmid pKC1139-trAI Cas9 is a fine-tuned CRISPR-Cas9 genome editing tool. It contains a TRAI-Cas9 cassette to precisely and efficiently remove genomic fragments and is suitable for genome editing of industrial *Streptomyces*. *cas9* expression is repressed under the non-induction condition and its highly expressed cognate inhibitor AcrIIA4. The high expression of Cas9 can be induced with thiostrepton and theophylline for genome editing (Jiang et al., 2021). To construct the plasmid pKC1139-trAI Cas9-*PKSII-melC* for deletion of *PKSII* (*orf3388*-*orf3390*, totally 2.5 kb) and *melC1/C2* (*orf0284*-*orf0285*, totally 1.4 kb), the fragments containing *PKSII* and *melC* spacers were inserted into the *Bam*HI/*Xba*I site of pKC1139-trAI cas9 plasmid. The left and right homologous fragments of *PKSII* were cloned by primers 1 and 2, primers 3 and 4, respectively. And the left and right homologous fragments of *melC* were amplified from L30 genomic DNA with primers 5 and 6, and primers 7 and 8, respectively. Then we inserted DNA fragments as above into the *Eco*RI site of pKC1139-trAI cas9-spacer plasmid with the ClonExpress MultiS One step Cloning Kit (Vazyme, China). Plasmid pSUC01 is a suicide plasmid containing the indigoidine synthetase gene from *S. albidoflavus* J1074 for efficient gene deletion. When the indigoidine synthetase gene exists after a single-crossover, the mutants of conjugants are blue with apramycin resistance. After multiple replicates with non-selection, the double-crossover mutants can be readily obtained with the loss of blue pigments and apramycin resistance (Dong et al., 2021). The plasmid pSUC01-*rppA* was used for the deletion of *rppA* (*orf0223*, 1.0 kb in total). The left and right homologous fragments of *rppA* were amplified from the L30 genomic DNA with primers 9 and 10, primers 11 and 12, respectively, and cloned into the *Eco*RI/*Kpn*I site of pSUC01. pKC1139 is a temperature-sensitive plasmid for gene deletion (Bierman et al., 1992). For construction of the pKC1139-*idgS-sfp* plasmid to insert *idgS-sfp* into the *dpt* gene cluster after *dptF*, the left and right homologous fragments of *dptF* were amplified with primers 13 and 14, primers 15 and 16, respectively, from the L30 genomic DNA, while the fragment of *idgS-sfp* was amplified with primers 17 and 18 from the plasmid pCIMt002 (Li et al., 2015), and cloned into the *Eco*RV/*Hin*dIII site of pKC1139. The fragment containing *neo* fragment was amplified from the plasmid pET28a with primers 19 and 20. The left and right homologous fragments of *dptD* were amplified from the L30 genomic DNA with primers 21 and 22, primers 23 and 24, respectively, and cloned into the *Eco*RI/*Xba*I site of pSUC01 for pSUC01-*neo* by the ClonExpress MultiS One step Cloning Kit (Vazyme, China). The PCR amplification conditions involved pre-denaturation at 94°C for 4 min, denaturation at 98°C for 10 min, annealing at 65°C for 15 s, and extension at 68°C (time was calculated according to 1 kb/min) with 30 cycles.

**TABLE 2:** Plasmids used in this study.

Plasmids	Description	Reference
pKC1139	*E.coli - Streptomyces* shuttle vector with pSG5 *ori*	Bierman et al. (1992)
pKC1139-trAI cas9-*PKSII-melC*	*PKSII* and *melC* knockout plasmid based on pKC1139	This study
pKC1139-*idgS-sfp*	*idgS-sfp in situ* insertion plasmid based on pKC1139	This study
pSUC01	Derived from pSET152 containing kasOp*-SaindC cassette	Dong et al. (2021)
pSUC01-*rppA*	*rppA* in-frame deletion plasmid based on pSUC01	This study
pSUC01-*neo*	*neo in situ* insertion plasmid based on pKC1139	This study
pCIMt002	Derived pBluescript II KS(+) containing ermEp*-idgS-sfp	Li et al. (2015)
pET28a	Protein expression vector containing *neo* gene	Novagen

**Strain construction for the dual reporter.** To avoid chromogenic interference, genes *PKSII*, *melC1/C2* (*melC*), and *rppA* were knocked out by in-frame deletion. The recombinant plasmid pKC1139-trAI cas9-*PKSII*-*melC* was transformed into ET12567/pUZ8002 and conjugated with *S. roseosporus* L30 wild-type (WT) strain. The recombinants were selected on the R5 agar medium containing 50 μg/mL apramycin. After culturing at 30°C for 5 days, the mycelia were transferred to the TSB liquid medium containing 15 μg/mL thiostrepton and 10 mM theophylline to induce the simultaneous deletion of *PKSII* and *melC*. The bacterial suspension was diluted 10^2^ and plated on the R5 agar plate. PCR was then performed to identify *PKSII*-*melC* double knock-out strains (Jiang et al., 2021)*.* The plasmid pSUC01-*rppA* was introduced into the *ΔPKSIIΔmelC* dual knockout strain by intergeneric conjugation. The recombinants were selected on the R5 agar medium containing 50 μg/mL apramycin and the blue single-crossover strains were transferred to a new R5 agar medium. After growth at 30°C for 3 days, the mycelia of blue strains were inoculated into the TSB liquid medium without apramycin, and the bacterial suspension was diluted 10^3^ and cultured on the R5 agar plate. The white double-crossover strains were picked and PCR was performed to identify the *ΔPKSIIΔrppAΔmelC* triple knock-out strains (Dong et al., 2021), which was designated L31. To insert promoter-less *neo* gene into the *dpt* gene cluster after *dptD* in the *ΔPKSIIΔrppAΔmelC* strain, the recombinant plasmid pSUC01-*neo* was conjugated into the triple knock-out strain through ET12567/pUZ8002. And the *neo* insertion mutant was developed as the same procedure to delete *rppA* described above. The white double-crossover strains were picked and PCR was performed to identify the *neo*-*ΔPKSIIΔrppAΔmelC* strain. Finally, the recombinant plasmid pKC1139-*idgS-sfp* was introduced into the strain from the previous step by ET12567/pUZ8002. The recombinants were selected on the R5 agar medium containing apramycin. After growth at 37°C for 5 days with 50 μg/mL apramycin, the mycelia from different single colonies were collected and PCR was performed to select single-crossover mutants, which were then inoculated on a new R5 agar medium without apramycin at 37°C for 3 days for double cross-over and plasmid loss. The spores were collected and spread to single colonies. The mycelia from different single colonies were then collected and PCR was performed to select double-crossover mutants with insertion of *idgS*-*sfp* fragment in the *neo*-*ΔPKSIIΔrppAΔmelC* strain into the *dpt* gene cluster after *dptF* (Sun et al., 2020). The dual-reporter system strain was designated L32.

**Optimization of UV-based random mutagenesis.** To test whether the medium was suitable for the dual-reporter strain (L32) and produce the blue pigment, mycelia of L32 were fermented in the TSB liquid medium for 2 days to reach the logarithmic growth, and an appropriate amount of bacterial solution was transferred on ISP4, YMG, MS, and R5 plates, and incubated at 30°C for 2 weeks. To test the Km resistance of the reporter strain before UV mutagenesis, mycelia of L32 were fermented in a shake flask with the liquid YEME medium. After the bacteria grew to the logarithmic growth phase, 20 μL of mycelia were spread on the YMG plates with Km gradient concentration from 0 to 50 μg/mL, and cultured at 30°C for 5 days to determine the minimum tolerance concentration of kanamycin after UV mutagenesis. Then UV radiation time by conducting a lethality curve and dilution of UV treated spores were optimized. The spores of the dual-reporter strain, which was incubated in TSB liquid medium for 2 days, were diluted with water to OD_600_ = 0.4–0.6, and exposed to ultraviolet radiations with the wavelength of 254 nm at a distance of 37 cm (8 W UV-Lamp) for 0, 1, 2, 3, 4, 5, 6, 7, and 8 min, respectively. Three parallel groups for each mutagenesis were set up. The UV treated spores were diluted with water to 100 and 1000 folds (1× UV treated spores as a control) and transferred to the R5 agar plate containing 50 μg/mL kanamycin and incubated in the dark, respectively. After 7 days, the number of colonies were counted to draw the lethality curve. Lethal rate (%) = (number of plate colonies before mutagenesis - number of plate colonies after mutagenesis)/number of plate colonies before mutagenesis × 100%. The dilution concentration with uniform distribution of single colonies and the appropriate number was used as the concentration standard for the next dilution. The time of UV radiation with a lethal rate of more than 90% was selected as the time standard for the next mutagenesis.

**Screening of random mutagenesis.** The spores of the reporter strain were exposed to ultraviolet radiations, and grown on R5 plates with 50 μg/mL Km, concentration for about 5 days in the dark. The single colonies with the dark blue in each plate were picked and inoculated in a YEME liquid medium for fermentation. 20 μL of the bacterial suspension was patched on the Km resistance gradient plates from 50 to 100 μg/mL and from 100 to 200 μg/mL km. At the same time, the starting reporter strain L32 without UV treatment was used as a control. Next, mutants with higher Km resistance than L32 obtained from the above mutagenesis were picked and fermented in a shake flask in the YEME medium. 1 mL of fermentation broth in 36, 48, 60, 72, 84, and 96 h, respectively, was collected to quantitatively evaluate the indigoidine production on high performance liquid chromatography (HPLC).

**Production and analysis of indigoidine and daptomycin.** In total, 1 mL of the fermentation broth was collected and terminated by adding an equal volume of methanol. The supernatant was harvested by centrifugation and analyzed on HPLC using a Shimadzu LC-20AP with an Agilent ZORBAX SB-C18 column (4.6 × 150 mm, 5 µm) (Agilent Technologies). The HPLC condition of daptomycin began with 10% acetonitrile for 5 min, followed by a linear gradient from 15% to 55% acetonitrile over 20 min, and subsequently, the condition was switched to 95% acetonitrile for 5 min and then restored to the initial composition of 10% acetonitrile for another 5 min. A constant flow rate of 0.5 mL/min was maintained. Detection of the analyte was performed at 215 nm with pure daptomycin as a standard. Detection of the analyte was performed at 215 nm with pure daptomycin as a standard. The HPLC condition of indigoidine began with 10% acetonitrile for 5 min, followed by a linear gradient from 10% to 50% acetonitrile over 30 min, the condition was then switched to 95% acetonitrile for 5 min, and then restored to the initial composition of 10% acetonitrile for another 5 min. A constant flow rate of 1 mL/min was maintained. Detection of the indigoidine was performed at 560 nm.

**HR-LC-MS analysis of indigoidine.** We collected 1 mL of the fermentation broth and terminated by adding an equal volume of methanol. The fermentation broth was centrifugated at 13,000 rpm at 4°C for 10 min. The supernatant was transferred to a new centrifuge tube and subjected to LC-MS on an Agilent Eclipse TC-C18 column (5 μm, 4.6 × 250 mm, Agilent Technologies), at a flow rate of 0.3 mL/min. Solution A (pure water) and solution B (pure acetonitrile) were employed to isolate the components with ultraviolet detection set at 560 nm. Elution was initiated with 10% acetonitrile for 5 min, then followed by a constant gradient from 10% to 55% acetonitrile over 10 min, and subsequently, the condition was switched to 95% acetonitrile for 5 min and then restored to the initial composition of 10% acetonitrile for another 5 min. The MS system was operated with an electrospray ionization source in positive ion mode.

**Preparation of *Streptomyces* RNA and real-time quantitative PCR (qRT-PCR)**. RNA was prepared from mycelia using an RN43-EASY spin Plus kit (China Aidlab Biotechnologies Co., Ltd.) to ensure the ratio of A_260_/A_280_ at 1.9–2.0. Genomic DNA was removed with RNase-free DNaseI (TaKaRa) and cDNA was prepared with HiScriptII Reverse Transcriptase (Vazyme). The reverse transcriptional reaction involves 50°C for 15 min and 85°C for 5 s. Then qRT-PCR was performed with R222-HiScriptII QRT SuperMix for qPCR (Vazyme) of genes *neo* (primers 27 and 28), *idgS-sfp* (primers 29 and 30), *dptA* (primers 31 and 32), *dptBC* (primers 33 and 34)*, dptD* (primers 35 and 36) and *dptE* (primers 37 and 38), with a house-keeping gene *orf0041* (primers 25 and 26) as an internal control. The qRT-PCR reaction procedures included 95°C for 30 s, 95°C for 5 s, 56°C for 5 s, and 72°C for 20 s. A total of 40 cycle numbers were taken from the second to the fourth step. The relative fold changes in gene expression were calculated based on the following formula 2^−ΔΔCT^.

## Results and discussion

### Development of a native-locus dual reporter system

Our previous work in *S. roseoporus* has developed a reporter system for the screening of a negative regulator on the *dptE* promoter (*dptEp*) (Luo et al., 2018). The kanamycin-resistant (Km^r^) gene *neo* was placed under *dptEp*, and ectopically integrated into the genome, while the transposon was used for random insertion mutagenesis. Though a negative regulator PhaR was eventually identified, the screening efficiency was not satisfactory, since the daptomycin production from most mutants was not significantly improved (Luo et al., 2018). It might result from the single but not global insertion for mutagenesis or the artificial Km resistance, possibly due to mutation on the Km target, or the chromosomal position effects (Baltz, 2009).

To bypass the genomic polar effects, here we designed a dual reporter system on the native daptomycin (*dpt*) gene cluster. To ensure the integrity of the giant transcript if the gene cluster is over-expressed and the correlation between daptomycin production and the reporters, the Km reporter gene *neo* was inserted after the last NRPS gene *dptD* as part of the giant operon. To avoid artificial Km resistance for more reliability of the reporter system, another chromogenic gene *idgS* was introduced. IdgS is a non-ribosomal peptidyl synthetase (NRPS) to produce a blue pigment indigoidine with Gln as the endogenous substrate, thus eliminating the requirement of exogenous addition of substrates, which might be uneven (Li et al., 2015). Here promoter-less *idgS* along with *Bacillus subtilis sfp* gene, which encodes a phosphopantetheinyl transferase (PPTase) to specifically activate IdgS in *Streptomyces* (Li et al., 2015), was integrated after *dptF* as a whole operon to form a dual reporter system along with *neo*. For comprehensive alterations and more convenient manipulation, UV-based random mutagenesis was conducted.

**FIGURE 1:**
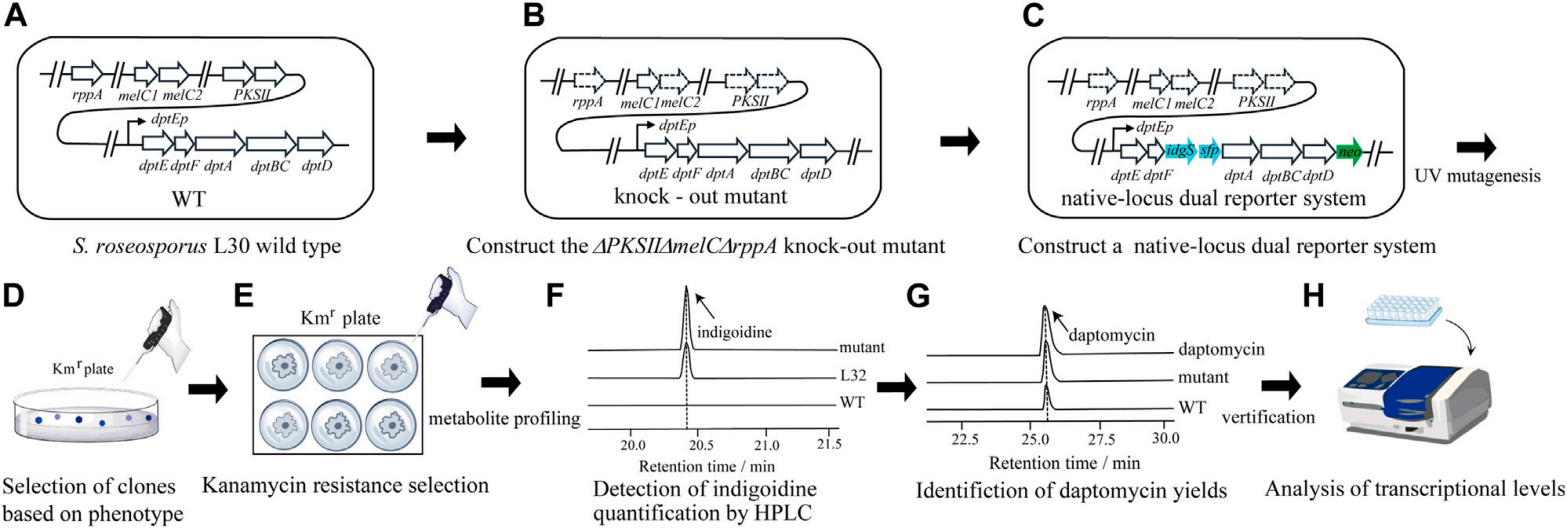
Workflow of the native-locus dual reporter system for screening of strains with improved yields of daptomycin in *S. roseosporus*. Based on the wild type strain L30 **(A)**, genes related to the endogenous pigment production were deleted to construct a triple mutant *ΔPKSIIΔmelCΔrppA* **(B)**. Then the Km resistant gene *neo* and indigoidine production gene *idgS* were integrated at the *dpt* gene cluster after *dptD* and *dptF*, respectively, to construct a native-locus dual reporter system **(C)**. Random mutagenesis was carried out under UV **(D)** and colonies were subject to simultaneous screening of Km resistance and bright blue **(E)**. Quantitative evaluation of indigoidine **(F)** and daptomycin production **(G)** was further taken to confirm the phenotypes. **(H)** Transcriptional expression of *neo* and *idgS* genes, as well as the daptomycin gene cluster, were examined to verify the correlation between improved productivity and gene expression.

*S. roseosporus* also produces a red pigment as one of the main secondary metabolites proposed from a type II polyketide synthase (PKSII) (Fang et al., 2022). Moreover, *Streptomyces* contains endogenous *rppA* and *melC1/C2* genes, which are highly conserved within this genus (Li et al., 2007; Lee et al., 2015). RppA is a type III chalcone synthase-related polyketide synthase and MelC is tyrosinase. Both enzymes are proposed to produce dark pigments, which might interfere with the effective screening from indigoidine produced by IdgS (Figure 1A). To avoid the chromogenic interference, the genes for PKSII (*PKSII*) and *rppA* were knocked out by in-frame deletion, followed by in-frame removal of *melC1/C2* genes (Supplementary Figures S1–3) (Figure 1B). Based on this triple knock-out strain, the promoter-less *neo* and *idgS-sfp* genes (Li et al., 2015) were sequentially inserted right after *dptD* and *dptF*, or right after and before the NRPS genes, respectively (Supplementary Figures S4, S5), as parts of the giant operon to form a native-locus dual reporter system (Figure 1C). Thus, we developed a reporter strain (L32) (*ΔPKSIIΔrppAΔmelC* + *neo* + *idgS*) for downstream mutagenesis and target screening.

### Optimization for UV-induced mutagenesis

**FIGURE 2:**
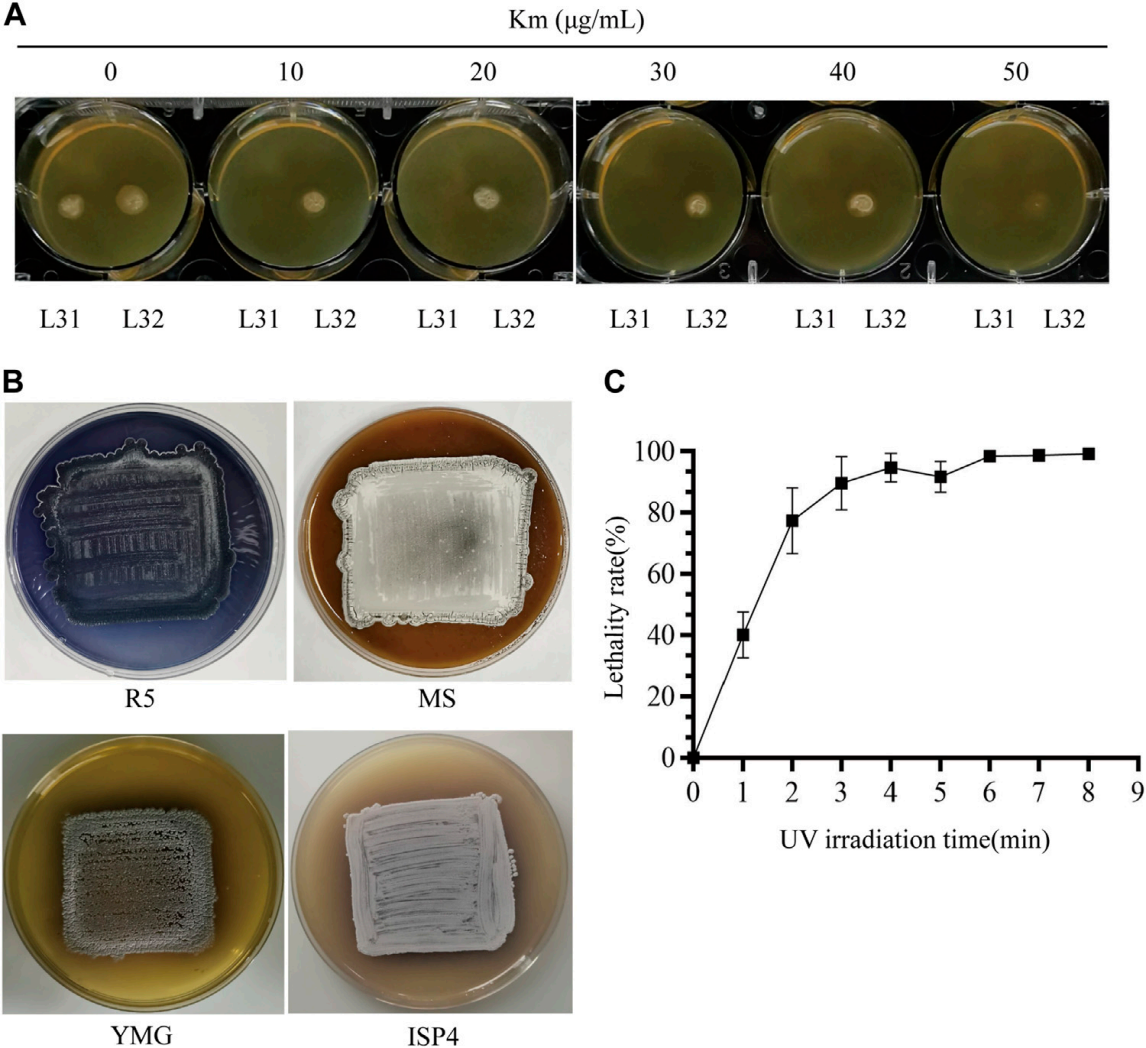
Condition optimization for random mutagenesis based on the native-locus dual reporter system. **(A)** Titration to determine Km resistance concentration for screening of the reporter system. Both the triple mutant (*ΔPKSIIΔmelCΔrppA*) (L31) and the reporter strain (L32) were patched on the YMG plates with increased concentration of Km from 0 to 50 μg/mL. **(B)** Phenotypes of the reporter strain on R5/MS/YMG/ISP4 agar plates. **(C)** Lethality curve after UV radiation. The error bars were produced by calculating the standard deviation (SD) of the data from three replicates of each sample.

Before we conducted UV-based mutagenesis and screened for strains ideally with increased Km resistance, *de novo* production of indigoidine and high production of daptomycin (Figures 1D–H), the initial condition of the starting reporter strain as well as the conditions for random mutagenesis were examined. After titration of the Km concentration, we found high sensitivity to Km from the triple mutant (*ΔPKSIIΔrppAΔmelC*) (L31), whose growth was completely inhibited at 10 μg/mL. However, the reporter strain (L32) consistently showed resistance to Km even up to 40 μg/mL, but almost no growth under 50 μg/mL Km, suggesting that the *dpt* gene cluster has moderate expression to drive *neo* transcription on the YMG medium (Figure 2A). Moreover, as a control, *S. roseosporus* L32 containing *idgS-sfp* under the native *dptEp* produced the blue pigment on the R5 medium, but not on MS, YMG or ISP4, suggesting that this chromogenic system can work in *S. roseosporus*. And the rich R5 medium was used for the downstream indigoidine production screening (Figure 2B). The most important parameter is the UV reagent to generate the appropriate lethality rate for *S. roseosporus*. With the spores on the dilution of 10^2^, under the UV light with a powder 8 W and 37 cm as the distance for different UV radiation time from 0 min to 8 min, this reporter strain showed over 90% lethality rate after exposure for 3 min, and about 95% lethality rate after exposure for over 6 min (Figure 2C). Based on these optimizations, the kanamycin resistance selection of the reporter strain after mutagenesis was higher than 50 μg/mL, and R5 was used as a medium suitable for the growth of the reporter strains for the subsequent determination of indigoidine pigment and daptomycin yield, and duration time of 3–8 min was used for the downstream UV mutagenesis.

### Target screening of the native-locus dual reporter system after UV-induced mutagenesis

Four independent rounds of UV radiation for mutagenesis were carried out under the conditions optimized above, and the randomly mutagenized cells were spread on the R5 medium initially with 50 μg/mL Km for screening after dilution of 10^2^. Among these colonies, 2, 5, 6, and 4 colonies were producing blue pigments, with the rate of 20%, 83%, 100%, and 100% for the correlated effectiveness of both reporters, respectively, after radiation for 5, 6, 7, and 8 min (Supplementary Figure S6), suggesting that UV radiation over 7 min is sufficient to ensure the high correlation of these two reporters. Simultaneous growth on the selection of 50 μg/mL Km and strong production of the blue pigment from these strains also suggested that both reporter genes *neo* and *idgS-sfp* should be expressed in higher levels, most probably resulting from the enhanced expression of the daptomycin gene cluster. Moreover, the high rate of the desired mutants (elevated Km resistance and blue pigment production) also suggested that our system is efficient for target screening.

**FIGURE 3:**
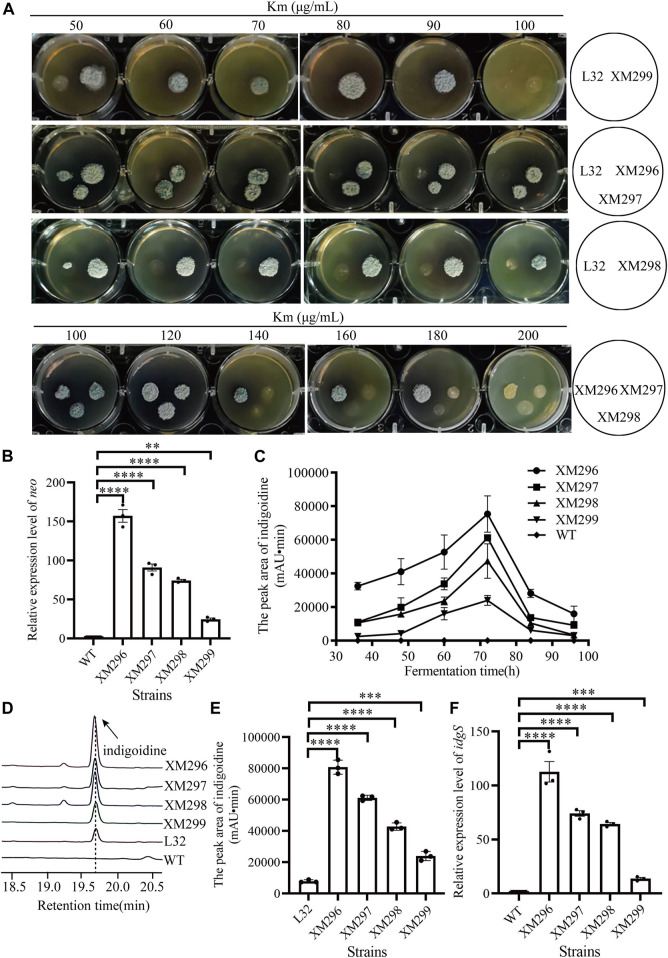
Screening of mutants based on the dual reporter system. **(A)** Test of Km resistance (50–200 μg/mL) of four mutants (XM296-XM299) after screening on 50 μg/mL Km, with the reporter strain L32 as a control. **(B)** Transcriptional analysis of *neo* gene by qRT-PCR in WT strain and four mutants from **(A)** at 72 h. **(C)** Production curve of indigoidine of the WT strain and four screened mutants. The error bars were produced by calculating the SD of the data from three replicates of each sample. **(D)** The relative production of indigoidine from the reporter strain and four mutants, examined on HPLC. The metabolite samples were collected at 72 h. **(E)** Quantitative assays of indigoidine production from HPLC. The samples of metabolites were collected at 72 h from the WT strain and four screened mutants. **(F)** Transcriptional assays of *idgS* gene by qRT-PCR in WT strain and the four mutants from **(A)** at 72 h. All the relative analysis data were presented as Mean ± SD (n = 3). One-way ANOVA with Dunnett’s *post hoc* test was used for statistical significance analysis. *** represents *p*-value < 0.001, and **** represents *p-*value < 0.0001.

Four mutants (XM299, XM298, XM297, and XM296) obtained from the above mutagenesis (with dark blue by visual inspection) were then further quantitatively evaluated. We found that the starting reporter strain L32 showed very poor growth on the YMG medium containing Km from 50 μg/mL to 100 μg/mL, while all four mutants grew very well, though XM299 showed reduced growth with 100 μg/mL Km (Figure 3A). Moreover, three mutants (XM298, XM297, and XM296) consistently showed apparent growth with Km from 100 μg/mL to 200 μg/mL, though XM298 and XM297 had reduced growth compared to XM296 after 120 μg/mL Km (Figure 3A), suggesting that XM296 might have the strongest *neo* expression than others. Consistent with these observations on plates, we found that all four mutants had significantly enhanced *neo* expression, and XM296 had the strongest *neo* expression (Figure 3B). The improved *neo* expression was well correlated with the resistance of these mutants to Km (Figure 3A).

The production of the blue pigment indigoidine was quantified. The presence of indigoidine in the reporter strain (L32) was confirmed by HR-LC-MS (Supplementary Figure S7). We found that all four mutants accumulated indigoidine during fermentation in the YEME liquid medium before 72 h, though the productivity dropped afterward (Figure 3C). Consistent with the growth advantage under Km selection, the mutant XM296 showed the highest indigoidine productivity throughout the fermentation phases, followed by strains XM297, XM298, and XM299. Further examination of indigoidine from fermentation at 72 h on HPLC showed that this blue pigment was almost undetectable in the wild type strain L30, while the starting reporter strain L32 could produce the blue pigment, as it also showed partial resistance to Km (Figure 2A), and all the mutants had much higher indigoidine productivity than the starter strain L32 (Figure 3D). Further quantitative assays showed that XM296−XM299 had enhanced production of 9.3, 6.8, 4.4, and 2.0 folds, respectively (Figure 3E). Real-time quantitative PCR assays also confirmed the improved gene expression of *idgS* in all four mutants to confer the enhanced production of indigoidine, while XM296 also showed the highest expression of *idgS* (Figure 3F).

All of the above data suggested that, based on our native-locus dual reporter system, we could efficiently obtain the mutants with significantly improved reporter effects, though the reporter output strength might vary among mutants.

### Hyper-production of daptomycin after mutagenesis and target screening

**FIGURE 4:**
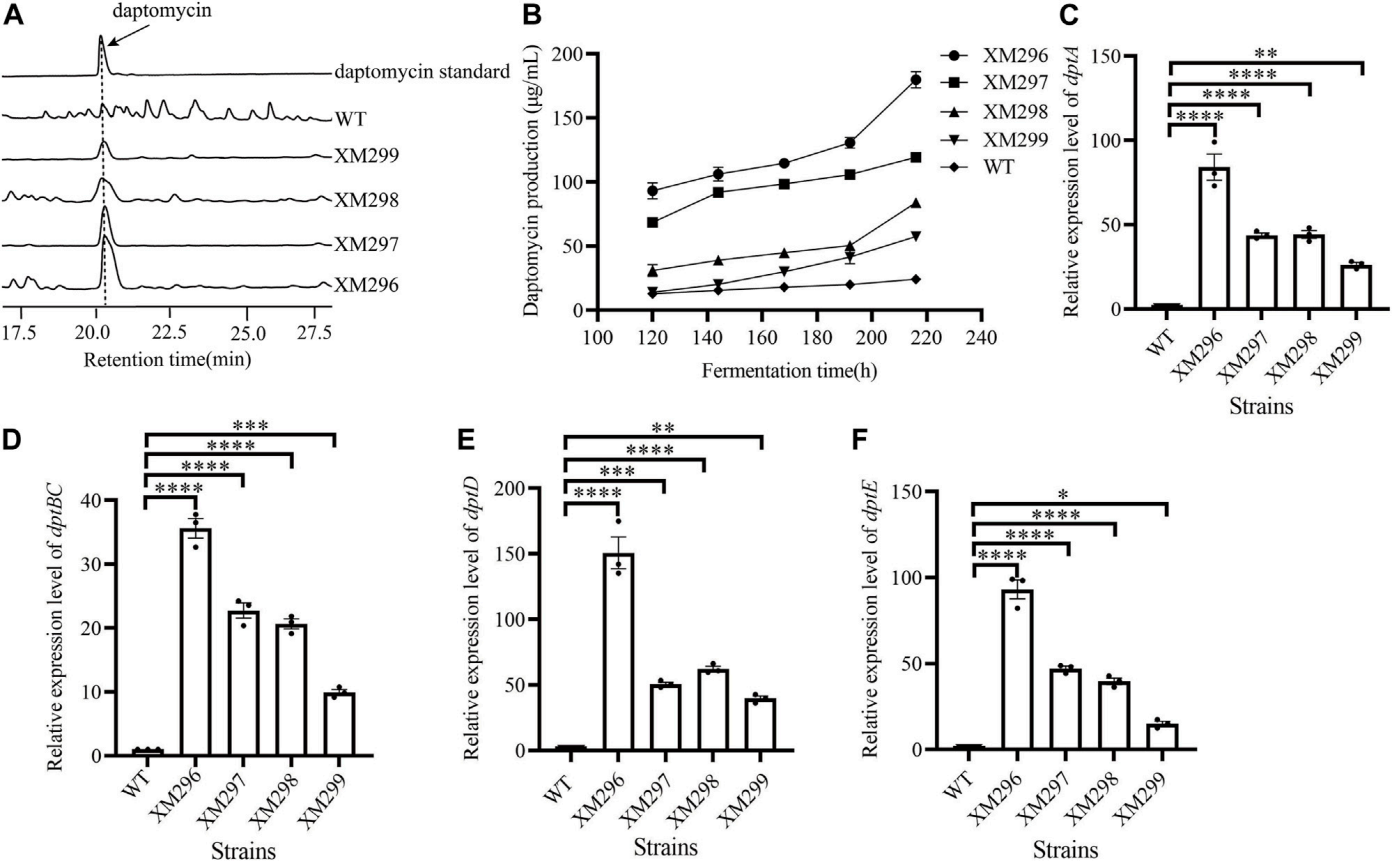
Metabolite and transcriptional analysis of daptomycin. **(A)** Representative metabolic profiling on HPLC of daptomycin production. The samples were collected at 216 h from four mutants, while the WT strain and daptomycin standard were used as controls. **(B)** The daptomycin production curves of four mutants and WT during the fermentation process. The metabolite samples were collected at 120, 144, 168, 192, and 216 h, respectively, and daptomycin production was analyzed on HPLC. The error bars were produced by calculating the SD of the data from three replicates of each sample. The relative transcriptional levels of *dptA* **(C)**, *dptBC* **(D)**, *dptD* **(E)**, and *dptE* **(F)** were examined using qRT-PCR from the WT strain and the four mutants. The mycelia samples in **(C**–**F)** were collected at 72 h. The error bars were produced by calculating the SD of the data from three replicates of each sample. One-way ANOVA with Dunnett’s *post hoc* test was used for statistical significance analysis. * represents the significant difference (*p*-value <0.05), ** represents *p*-value < 0.01, *** represents *p*-value < 0.001, and **** represents *p-*value < 0.0001.

Since both *neo* and *idgS* genes were integrated at the *dpt* native gene cluster as a whole operon, the simultaneously improved expression of *neo* and *idgS* genes in the mutants should result from the enhanced activity of the promoter *dptEp*, which controls the expression of the whole operon. Our data confirmed increased production of daptomycin in all four mutants (Figure 4A). The wild type strain showed a productivity of less than 25 mg/L throughout the fermentation. Strains XM298 and XM299 showed moderate improvement in daptomycin production, while a more remarkable increase in daptomycin production was observed in strains XM296 and XM297, consistent with their phenotypes of Km resistance and indigoidine production. In particular, strains XM296-XM299 showed 179.99, 119.46, 84.09, and 57.66 mg/L of daptomycin productivity at 216 h, which were 7.4, 4.9, 3.5, and 2.4 folds of wild type, respectively (Figures 4A,B). These four mutants consistently had significantly increased expression of core genes including *dptA*, *dptBC,* and *dptD*, which encode the non-ribosomal peptidyl synthetases (NRPSs) for the assembly of amino acids, as well as *dptE*, which is the first gene within the operon and encodes the acyl-CoA ligase for decanoic acid activation. Strain XM296 again showed the most prominent enhancement of gene expression among four mutants (Figures 4C–F). All these data supported the conclusion that the daptomycin production in these mutants was improved effectively, and well correlated with our native-locus dual reporter.

Our data showed that the strain XM296 had the most desired phenotypes, including the strongest Km resistance, as well as the highest productivity of indigoidine and 6.4-fold increased production of daptomycin, suggesting that this strategy would be very convenient and efficient for the hyper-production of natural products in *Streptomyces*. Moreover, it would also be very promising to obtain mutants with further improved productivity through other mutagenesis measures, such as ARTP, under our native-locus dual reporter system.

## Conclusion

For the efficient screening of the desired mutants after random mutagenesis for the hyper-production of natural products in *Streptomyces*, the present study developed a native-locus dual reporter system by integrating both the antibiotic-resistant gene *neo* and the endogenous chromogenic gene *idgS* at the native gene cluster for the biosynthesis of natural products (eg. daptomycin), ideally fused with backbone genes as a whole operon. Compared to the traditional single reporter system, the desired mutants could be readily obtained based on our dual reporter. The false positives could be significantly reduced, and the productivity was highly correlated with the effectiveness of both reporters. Moreover, the productivity of the target natural product can also be remarkably improved. The increased folds from our one-dose mutagenesis are comparable to the time-consuming strategy based on mechanism deciphering and network refactoring. This strategy could be widely and readily applied in *Streptomyces* to efficiently ramp up the productivity of drugs and activate the cryptic gene clusters, to speed up the pharmaceutical development in *Streptomyces*.

## Data Availability

The original contributions presented in the study are included in the article/Supplementary Material, further inquiries can be directed to the corresponding authors.

## References

[B1] Baltz R. H. (2009). Biosynthesis and genetic engineering of lipopeptides in *Streptomyces roseosporus* . Methods Enzymol. 458, 511–531. 10.1016/S0076-6879(09)04820-4 19374996

[B2] Bierman M. Logan R. O'Brien K. Seno E. T. Rao R. N. Schoner B. E. (1992). Plasmid cloning vectors for the conjugal transfer of DNA from *Escherichia coli* to *Streptomyces* spp. Gene 116, 43–49. 10.1016/0378-1119(92)90627-2 1628843

[B3] Bilyk B. Horbal L. Luzhetskyy A. (2017). Chromosomal position effect influences the heterologous expression of genes and biosynthetic gene clusters in *Streptomyces albus* J1074. Microb. Cell Fact. 16, 5. 10.1186/s12934-016-0619-z 28052753 PMC5209838

[B4] Dong J. Wei J. Li H. Zhao S. Guan W. (2021). An efficient markerless deletion system suitable for the industrial strains of *Streptomyces* . J. Microbiol. Biotechnol. 31, 1722–1731. 10.4014/jmb.2106.06083 34489377 PMC9705919

[B5] Fang J. L. Gao W. L. Xu W. F. Lyu Z. Y. Ma L. Luo S. (2022). m4C DNA methylation regulates biosynthesis of daptomycin in *Streptomyces roseosporus* L30. Synth. Syst. Biotechnol. 7, 1013–1023. 10.1016/j.synbio.2022.06.001 35801092 PMC9240718

[B6] Gongerowska-Jac M. Szafran M. J. Jakimowicz D. (2021). Combining transposon mutagenesis and reporter genes to identify novel regulators of the *topA* promoter in *Streptomyces* . Microb. Cell Fact. 20, 99. 10.1186/s12934-021-01590-7 33985526 PMC8120823

[B7] Hertweck C. (2009). The biosynthetic logic of polyketide diversity. Angew. Chem. Int. Ed. Engl. 48, 4688–4716. 10.1002/anie.200806121 19514004

[B8] Jiang Y. H. Liu Y. F. Wang K. Zhou J. Y. Guo F. Zhao Q. W. (2021). Fine-tuning Cas9 activity with a cognate inhibitor AcrIIA4 to improve genome editing in *Streptomyces* . ACS Synth. Biol. 10, 2833–2841. 10.1021/acssynbio.1c00141 34734710

[B9] Kang H. S. Kim E. S. (2021). Recent advances in heterologous expression of natural product biosynthetic gene clusters in *Streptomyces* hosts. Curr. Opin. Biotechnol. 69, 118–127. 10.1016/j.copbio.2020.12.016 33445072

[B10] Khushboo M. T. Kumar P. Rajput D. Yadav V. Dhaka N. Shukla R. (2023). Genome-guided approaches and evaluation of the strategies to influence bioprocessing assisted morphological engineering of *Streptomyces* cell factories. Bioresour. Technol. 376, 128836. 10.1016/j.biortech.2023.128836 36898554

[B11] Lee N. Lee S. H. Baek K. Kim B. G. (2015). Heterologous expression of tyrosinase (MelC2) from *Streptomyces avermitilis* MA4680 in *E. coli* and its application for ortho-hydroxylation of resveratrol to produce piceatannol. Appl. Microbiol. Biotechnol. 99, 7915–7924. 10.1007/s00253-015-6691-1 26036705

[B12] Li P. Guo Z. Tang W. Chen Y. (2018). Activation of three natural product biosynthetic gene clusters from *Streptomyces lavendulae* CGMCC 4.1386 by a reporter-guided strategy. Synth. Syst. Biotechnol. 3, 254–260. 10.1016/j.synbio.2018.10.010 30417141 PMC6223227

[B13] Li P. Li J. Guo Z. Tang W. Han J. Meng X. (2015). An efficient blue-white screening based gene inactivation system for *Streptomyces* . Appl. Microbiol. Biotechnol. 99, 1923–1933. 10.1007/s00253-014-6369-0 25666782

[B14] Li S. Gruschow S. Dordick J. S. Sherman D. H. (2007). Molecular analysis of the role of tyrosine 224 in the active site of *Streptomyces coelicolor* RppA, a bacterial type III polyketide synthase. J. Biol. Chem. 282, 12765–12772. 10.1074/jbc.m700393200 17331946

[B15] Liu R. Deng Z. Liu T. (2018). *Streptomyces* species: Ideal chassis for natural product discovery and overproduction. Metab. Eng. 50, 74–84. 10.1016/j.ymben.2018.05.015 29852270

[B16] Liu Z. Zhao Y. Huang C. Luo Y. (2021). Recent advances in silent gene cluster activation in *Streptomyces* . Front. Bioeng. Biotechnol. 9, 632230. 10.3389/fbioe.2021.632230 33681170 PMC7930741

[B17] Luo S. Chen X. A. Mao X. M. Li Y. Q. (2018). Transposon-based identification of a negative regulator for the antibiotic hyper-production in *Streptomyces* . Appl. Microbiol. Biotechnol. 102, 6581–6592. 10.1007/s00253-018-9103-5 29876602

[B18] Lyu Z. Y. Bu Q. T. Fang J. L. Zhu C. Y. Xu W. F. Ma L. (2022). Improving the yield and quality of daptomycin in *Streptomyces roseosporus* by multilevel metabolic engineering. Front. Microbiol. 13, 872397. 10.3389/fmicb.2022.872397 35509317 PMC9058172

[B19] Mao X. M. Luo S. Li Y. Q. (2017). Negative regulation of daptomycin production by DepR2, an ArsR-family transcriptional factor. J. Ind. Microbiol. Biotechnol. 44, 1653–1658. 10.1007/s10295-017-1983-3 29038911

[B20] Mao X. M. Luo S. Zhou R. C. Wang F. Yu P. Sun N. (2015). Transcriptional regulation of the daptomycin gene cluster in *Streptomyces roseosporus* by an autoregulator, AtrA. J. Biol. Chem. 290, 7992–8001. 10.1074/jbc.m114.608273 25648897 PMC4367297

[B21] Miao V. Coeffet-LeGal M. F. Brian P. Brost R. Penn J. Whiting A. (2005). Daptomycin biosynthesis in *Streptomyces roseosporus*: Cloning and analysis of the gene cluster and revision of peptide stereochemistry. Microbiol. Read. 151, 1507–1523. 10.1099/mic.0.27757-0 15870461

[B22] Mo S. Lee S. K. Jin Y. Y. Oh C. H. Suh J. W. (2013). Application of a combined approach involving classical random mutagenesis and metabolic engineering to enhance FK506 production in *Streptomyces* sp. RM7011. Appl. Microbiol. Biotechnol. 97, 3053–3062. 10.1007/s00253-012-4413-5 23053074

[B23] Moore S. J. Lai H. E. Li J. Freemont P. S. (2023). *Streptomyces* cell-free systems for natural product discovery and engineering. Nat. Prod. Rep. 40, 228–236. 10.1039/d2np00057a 36341536 PMC9945932

[B24] Nepal K. K. Wang G. (2019). *Streptomycetes*: Surrogate hosts for the genetic manipulation of biosynthetic gene clusters and production of natural products. Biotechnol. Adv. 37, 1–20. 10.1016/j.biotechadv.2018.10.003 30312648 PMC6343487

[B25] Nicault M. Tidjani A. R. Gauthier A. Dumarcay S. Gelhaye E. Bontemps C. (2020). Mining the biosynthetic potential for specialized metabolism of a *Streptomyces* soil community. Antibiot. (Basel) 9, 271. 10.3390/antibiotics9050271 PMC7277575 32456220

[B26] Onaka H. (2017). Novel antibiotic screening methods to awaken silent or cryptic secondary metabolic pathways in actinomycetes. J. Antibiot. (Tokyo) 70, 865–870. 10.1038/ja.2017.51 28442735

[B27] Quinn G. A. Banat A. M. Abdelhameed A. M. Banat I. M. (2020). *Streptomyces* from traditional medicine: Sources of new innovations in antibiotic discovery. J. Med. Microbiol. 69, 1040–1048. 10.1099/jmm.0.001232 32692643 PMC7642979

[B28] Rigali S. Anderssen S. Naome A. van Wezel G. P. (2018). Cracking the regulatory code of biosynthetic gene clusters as a strategy for natural product discovery. Biochem. Pharmacol. 153, 24–34. 10.1016/j.bcp.2018.01.007 29309762

[B29] Rubio S. Martinez-Camara S. de la Fuente J. L. Rodriguez-Saiz M. Barredo J. L. (2021). Strain improvement program of *Streptomyces roseosporus* for daptomycin production. Methods Mol. Biol. 2296, 351–363. 10.1007/978-1-0716-1358-0_20 33977458

[B30] Sun C. F. Xu W. F. Zhao Q. W. Luo S. Chen X. A. Li Y. Q. (2020). Crotonylation of key metabolic enzymes regulates carbon catabolite repression in *Streptomyces roseosporus* . Commun. Biol. 3, 192. 10.1038/s42003-020-0924-2 32332843 PMC7181814

[B31] Walsh C. T. (2016). Insights into the chemical logic and enzymatic machinery of NRPS assembly lines. Nat. Prod. Rep. 33, 127–135. 10.1039/c5np00035a 26175103

[B32] Xiang S. H. Li J. Yin H. Zheng J. T. Yang X. Wang H. B. (2009). Application of a double-reporter-guided mutant selection method to improve clavulanic acid production in *Streptomyces clavuligerus* . Metab. Eng. 11, 310–318. 10.1016/j.ymben.2009.06.003 19584003

[B33] Xu M. Wright G. D. (2019). Heterologous expression-facilitated natural products' discovery in actinomycetes. J. Ind. Microbiol. Biotechnol. 46, 415–431. 10.1007/s10295-018-2097-2 30446891

[B34] Xu W. F. Sun C. F. Gao W. L. Scharf D. H. Zhu C. Y. Bu Q. T. (2023). Degradation mechanism of AtrA mediated by ClpXP and its application in daptomycin production in *Streptomyces roseosporus* . Protein Sci. 32, e4617. 10.1002/pro.4617 36882943 PMC10031807

[B35] Xu Z. Wang Y. Chater K. F. Ou H. Y. Xu H. H. Deng Z. (2017). Large-scale transposition mutagenesis of *Streptomyces coelicolor* identifies hundreds of genes influencing antibiotic biosynthesis. Appl. Environ. Microbiol. 83, 028899–e2916. 10.1128/aem.02889-16 PMC5335527 28062460

[B36] Yan L. Zhang Z. Zhang Y. Yang H. Qiu G. Wang D. (2021). Improvement of tacrolimus production in *Streptomyces tsukubaensis* by mutagenesis and optimization of fermentation medium using Plackett-Burman design combined with response surface methodology. Biotechnol. Lett. 43, 1765–1778. 10.1007/s10529-021-03144-8 34021830

[B37] Ye L. Ye R. Hu F. Wang G. (2021). Combination of atmospheric and room temperature plasma (ARTP) mutagenesis, genome shuffling and dimethyl sulfoxide (DMSO) feeding to improve FK506 production in *Streptomyces tsukubaensis* . Biotechnol. Lett. 43, 1809–1820. 10.1007/s10529-021-03154-6 34160747

[B38] Yu Z. Lv H. Wu Y. Wei T. Yang S. Ju D. (2019a). Enhancement of FK520 production in *Streptomyces hygroscopicus* by combining traditional mutagenesis with metabolic engineering. Appl. Microbiol. Biotechnol. 103, 9593–9606. 10.1007/s00253-019-10192-8 31713669

[B39] Yu Z. Shen X. Wu Y. Yang S. Ju D. Chen S. (2019b). Enhancement of ascomycin production via a combination of atmospheric and room temperature plasma mutagenesis in *Streptomyces hygroscopicus* and medium optimization. Amb. Express 9, 25. 10.1186/s13568-019-0749-x 30778695 PMC6379505

