# Supplementary material for: Development of a native-locus dual reporter system for the efficient screening of the hyper-production of natural products in Streptomyces
Source: Front Bioeng Biotechnol. 2023 Jun 29;11:1225849. doi: 10.3389/fbioe.2023.1225849 (PMC10343952; doi:10.3389/fbioe.2023.1225849)
Supplement: Supplementary file 1 [file DataSheet1.pdf]

Supplementary Material

Development of a Native-locus Dual Reporter System for Efficient Screening of Hyper-production of Natural Products in *Streptomyces*

Jing-Yi Zhou[1,2#], Bin-Bin Ma[1,2#], Qing-Wei Zhao[1,3*], Xu-Ming Mao[1,2*]

[1]Department of Clinical Pharmacy, the First Affiliated Hospital & Institute of Pharmaceutical Biotechnology, School of Medicine, Zhejiang University, Hangzhou 310058, China

[2]Zhejiang Provincial Key Laboratory for Microbial Biochemistry and Metabolic Engineering, Hangzhou 310058, China

[3]Zhejiang Provincial Key Laboratory for Drug Evaluation and Clinical Research, Hangzhou 310006, China

[#] These authors contributed equally to this work and share the first authorship

*** Correspondence:**
Corresponding Author: qwzhao@zju.edu.cn (Q.-W.Z.); xmmao@zju.edu.cn (X.-M.M.)

Supplementary Figure 1. Deletion of gene *PKSII in S. roseosporus.* PCR was performed to confirm deletion of *PKSII* by primers on the upstream (A) and downstream (B) homologous fragments. (C) Diagramic illustration for *PKSII* deletion. The primers for diagnostic PCR were shown in arrows. The predicted sizes of PCR fragments were shown.

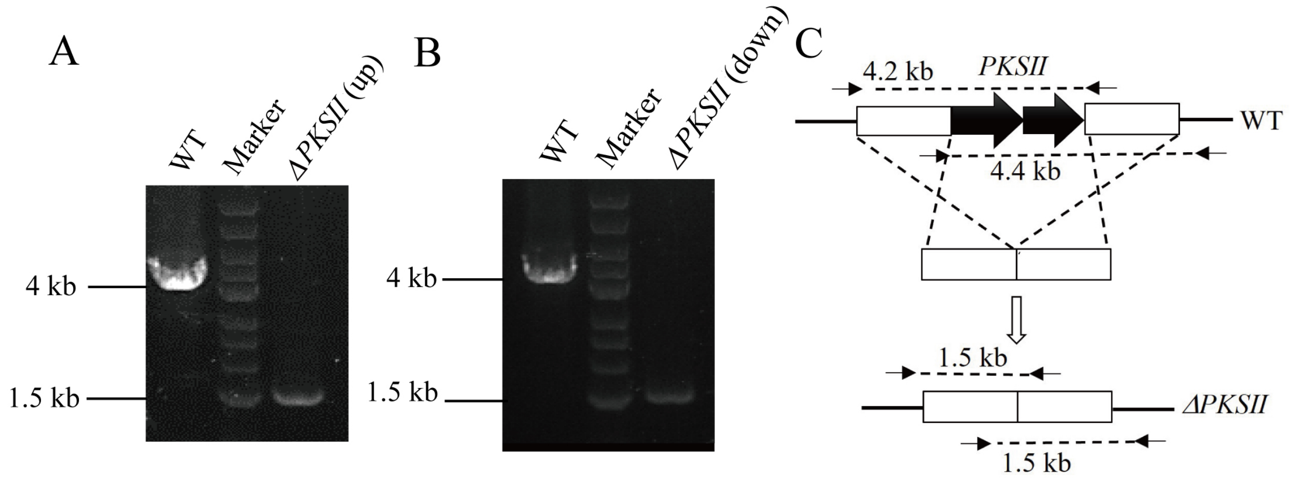

Supplementary Figure 2. Deletion of gene *melC* in the *ΔPKSII* mutant. PCR was performed to confirm deletion of *melC1/C2* (*melC*) by primers on the upstream (A) and downstream (B) homologous fragments. (C) Diagramic illustration for *melC* deletion. The primers for diagnostic PCR were shown in arrows. The predicted sizes of PCR fragments were shown.

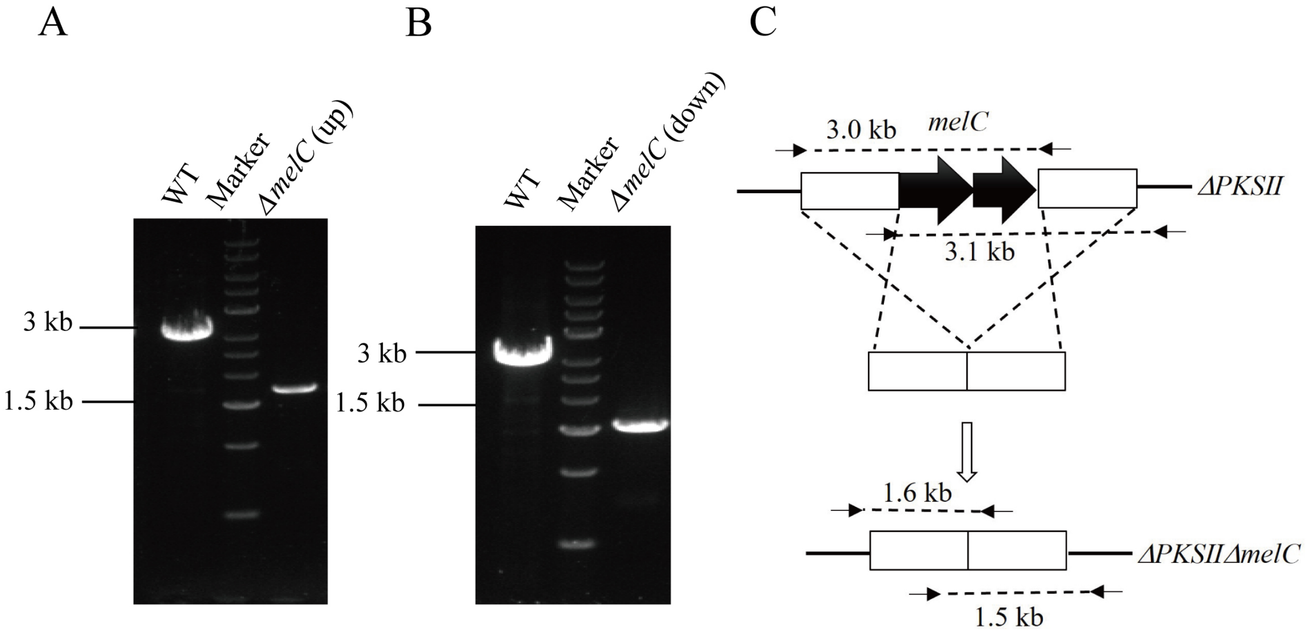

Supplementary Figure 3. Deletion of gene *rppA* in the *ΔPKSIIΔmelC* mutant. PCR was performed to confirm deletion of *rppA* by primers on the upstream (A) and downstream (B) homologous fragments. (C) Diagramic illustration for *rppA* deletion. The primers for diagnostic PCR were shown in arrows. The predicted sizes of PCR fragments were shown.

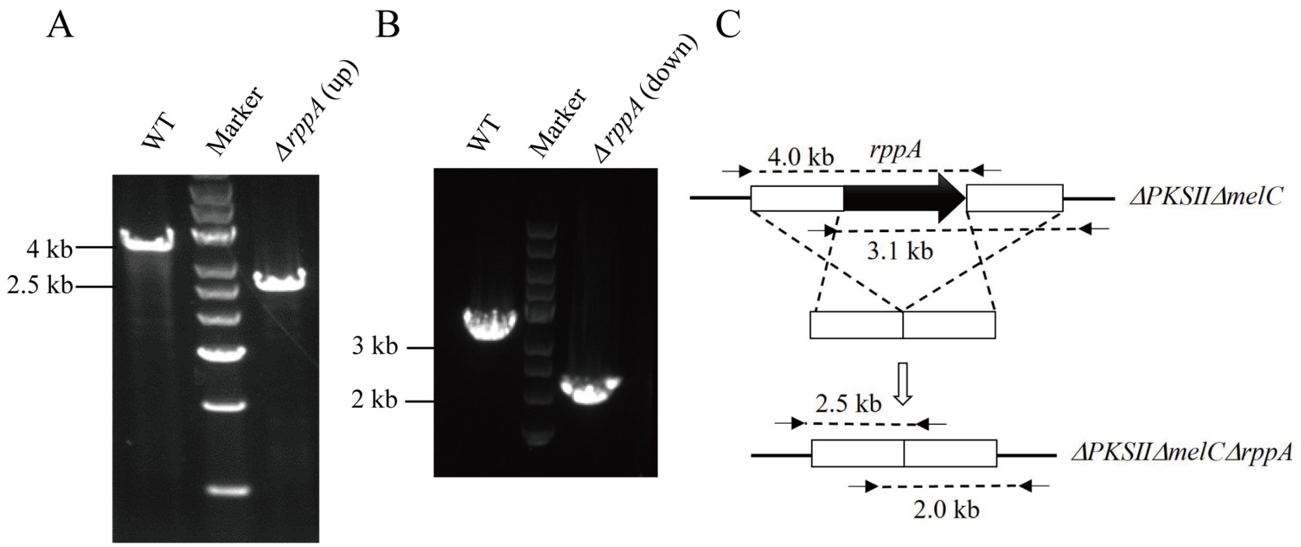

Supplementary Figure 4. Insertion of the reporter gene *neo* in the *ΔPKSIIΔmelCΔrppA* mutant. PCR was performed to confirm insertion of *neo* by primers on the upstream (A) and downstream (B) homologous fragments. (C) Diagramic illustration for insertion of *neo* right after *dptD*. The primers for diagnostic PCR were shown in arrows. The predicted sizes of PCR fragments were shown.

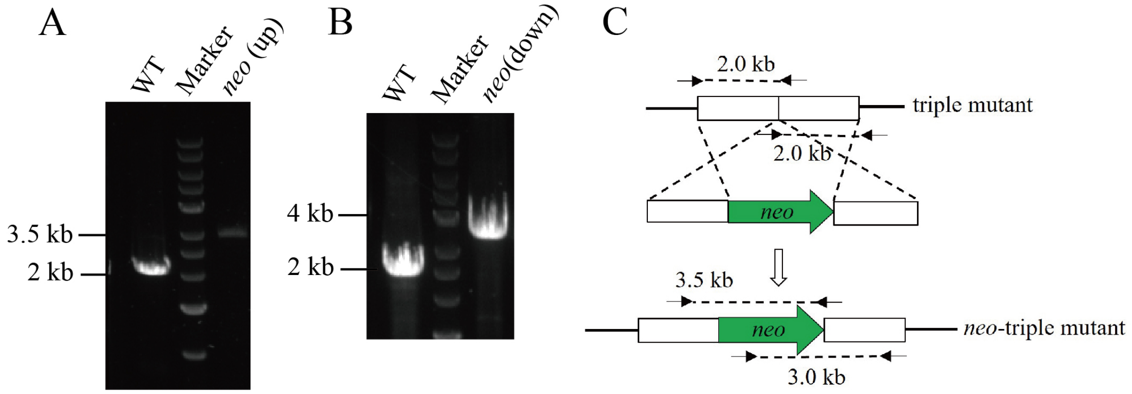

Supplementary Figure 5. Insertion of reporter gene *idgS-sfp* in the *ΔPKSIIΔmelCΔrppA* + *neo* strain. PCR was performed to confirm insertion of *neo* by primers on the upstream (A) and downstream (B) homologous fragments. (C) Diagramic illustration for insertion of *idgS-sfp* right after *dptF.* The primers for diagnostic PCR were shown in arrows. The predicted sizes of PCR fragments were shown.

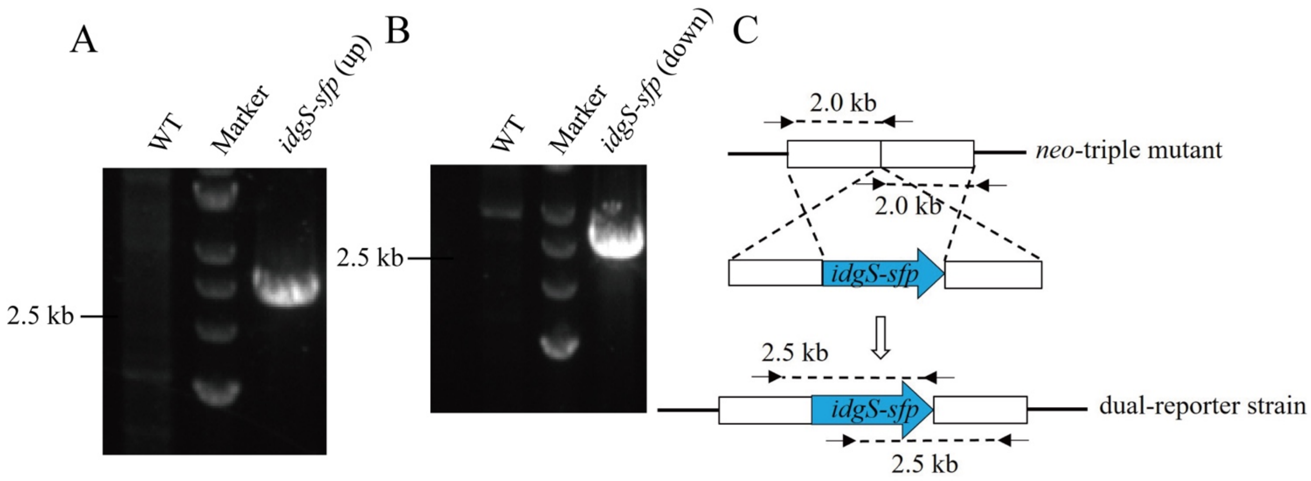

Supplementary Figure 6. Screening of mutants after UV radiation. The spores of the reporter strain L32 was exposed to UV for 5 (A), 6 (B), 7 (C) and 8 (D) min, respectively, and grown on the R5 medium with 50 μg/mL Km for 7 days, followed by photographing.

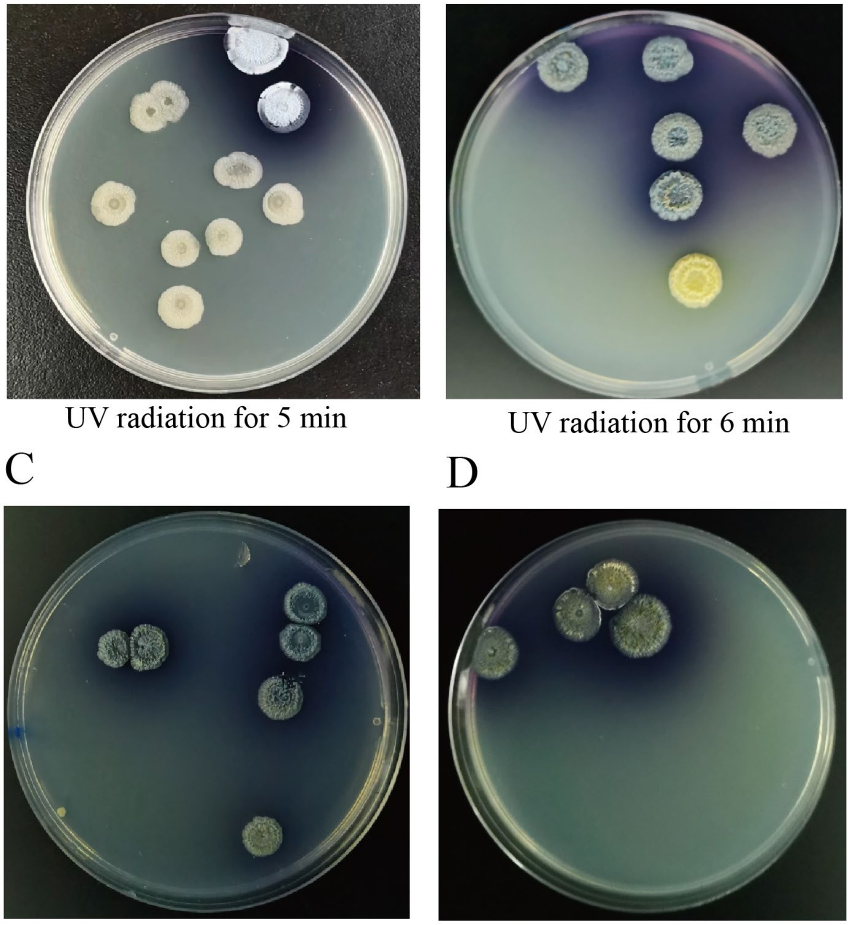

A UV radiation for 5 min

B UV radiation for 6 min

C UV radiation for 7 min

D UV radiation for 8 min

Supplementary Figure 7. Identification of indigoidine in fermentation broth. (A) HPLC analysis, (B) UV absorption spectrum and (C) LC-MS result of blue pigment collected from fermentation broth of native-locus dual reporter system strains after fermenting 72 h. The blue pigment has same characteristics above as the indigoidine that has been reported.

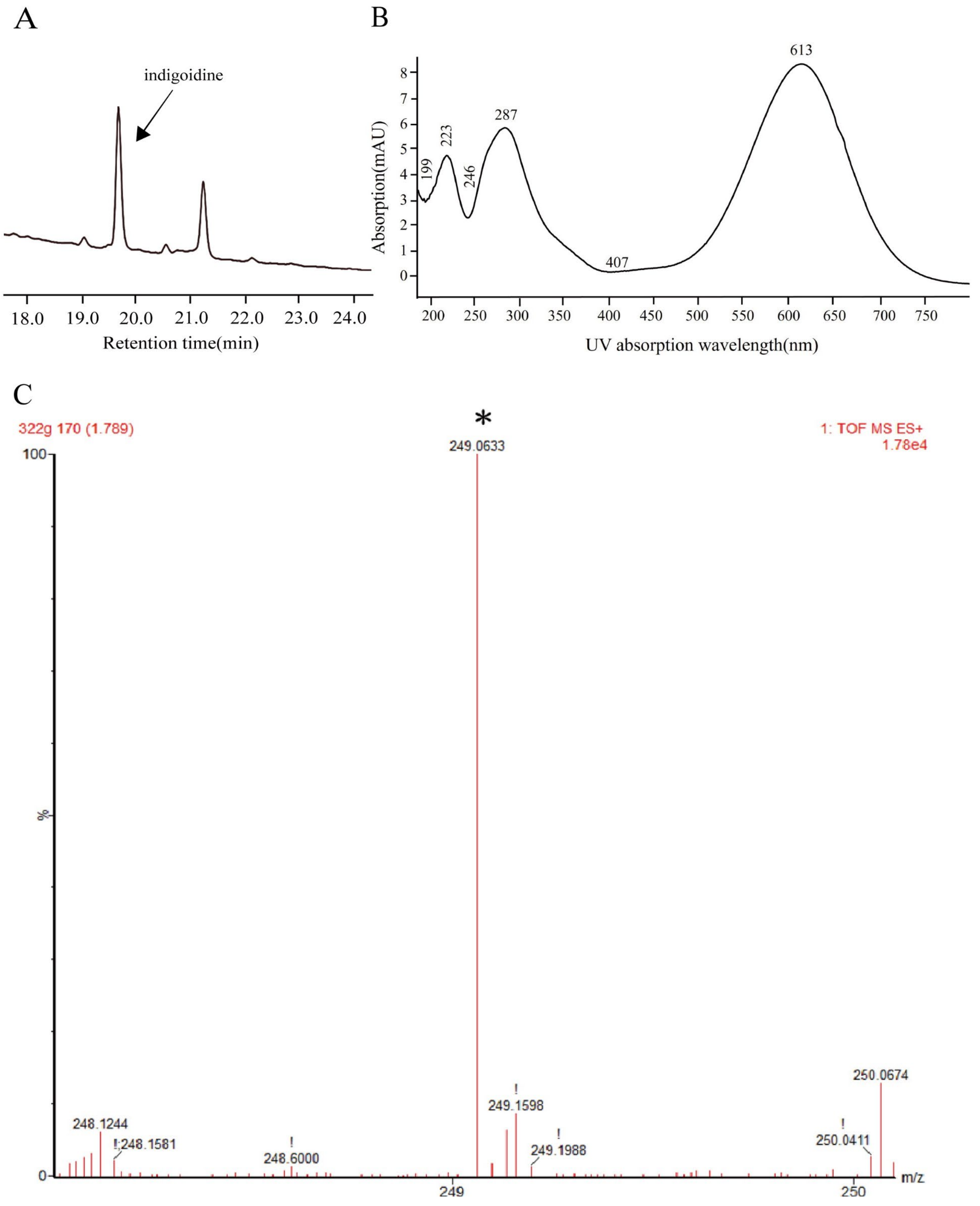

Supplementary Table 1. Primers used in this study

Plasmids	No	Sequence
pKC1139-trAI cas9-*PKSII-melC*	1	CGAGGACGACATCACGCC
	2	CCGCTCCGCACTCACCGG
	3	CCGTACCGAACCCAGGGC
	4	ACGGTGCGCAGGTGCTGGT
	5	CAGCAGCGAGAAGAAGGC
	6	GGCTCATGAGATGACTA
	7	TCTACCGCTACGCCTGAGA
	8	TCAACCCCGAGCATCTG
pSUC01-*rppA*	9	CTCTTCGCCTAGGAATTCGTCGAGGTGAACTCC
	10	CCTACCAGGTCATCCTGCCT
	11	GGATGACCTGGTAGGAAGGAAC
	12	CTAGACCATGGGGTACCTAGACGAGCAGCACC
pKC1139-*idgS-sfp*	13	CCAGTGCCAAGCTTGCTCTCATCCAGTACACCT
	14	GTAATTTCTCCTTCAGGTGCGGTCGGCCAA
	15	TAAGGGGATCCGCGAGAGATGGACATGCAGTC
	16	CATGATTACGAATTCGATATCCGACGACGTCCTCG
	17	CACCTGAAGGAGAAATTACATATGACTCTTCA
	18	ATCTCTCGCGGATCCCCTTATAAAAGCTCTTCG
pSUC01-*neo*	19	ACGAAAGAAAGGAGGCGGACATATGAGCCAT

	20	TTGGCCATGGCGGTATGTTTCTTAGAAAAACT
	21	AATTCAAGCTTGGATCCATCACGCACGTCA
	22	TGTCCGCCTCCTTTCTTTCGTGTACGGACA
	23	ATGAGTTTTTCTAAGAAACATACCGCCAT
	24	TCTAGACCATGGTCATGGTTTGCGTCCGTG
qRT-PCR control	25	GAAGACGACACGTGGAC
	26	CGTCGTCGTGAACAACGT
qRT-PCR *neo*	27	TGATGACGAGCGTAATG
	28	CTGGTATCGGTCTGCGAT
qRT-PCR *idgS*	29	AATCGACGTCGATCTGG
	30	TAACCGTTGCTGTTCTCG
qRT-PCR *dptA*	31	CCTCGATCACCTCCCTGT
	32	AGGATCCGTACGTGCTC
qRT-PCR *dptBC*	33	GTGAGGTGAAGGAGACTC
	34	GTGGTGGTTTCGTCGGTT
qRT-PCR *dptD*	35	CGATGAAGGACGTCTTCC
	36	GCAGTCGATCAGGTGCAG
qRT-PCR *dptE*	37	GCTTGATGGTGGTCCAC
	38	GTGGACGATGTTCAGCT
